# PCR Detection of Epstein-Barr Virus (EBV) DNA in Patients with Head and Neck Squamous Cell Carcinoma, in Patients with Chronic Tonsillitis, and in Healthy Individuals

**DOI:** 10.1155/2022/8506242

**Published:** 2022-08-08

**Authors:** Joanna Katarzyna Strzelczyk, Agata Świętek, Krzysztof Biernacki, Karolina Gołąbek, Jadwiga Gaździcka, Katarzyna Miśkiewicz-Orczyk, Wojciech Ścierski, Janusz Strzelczyk, Rafał Fiolka, Maciej Misiołek

**Affiliations:** ^1^Department of Medical and Molecular Biology, Faculty of Medical Sciences in Zabrze, Medical University of Silesia in Katowice, 19 Jordana St., 41-808 Zabrze, Poland; ^2^Department of Otorhinolaryngology and Oncological Laryngology, Faculty of Medical Sciences in Zabrze, Medical University of Silesia in Katowice, 10 C Skłodowskiej St., 41-800 Zabrze, Poland; ^3^Department of Endocrinology and Neuroendocrine Tumors, Department of Pathophysiology and Endocrinology, Faculty of Medical Sciences in Zabrze, Medical University of Silesia in Katowice, 35 Ceglana St., 40-514 Katowice, Poland; ^4^Doctoral School, Faculty of Medical Sciences in Zabrze, Medical University of Silesia in Katowice, 40-055 Katowice, Poland

## Abstract

Epstein-Barr virus (EBV) is a common virus worldwide that is an etiologic agent in the development of many diseases, including cancer. Recent reports have shown the association of EBV with tumorigenesis in head and neck squamous cell carcinoma (HNSCC). Moreover, EBV has been reported to be present in tonsillar tissues, which suggests a close relationship between viral infections and tonsillar diseases, including chronic tonsillitis. The aim of the study was to analyze the prevalence of EBV DNA in 86 patients with HNSCC, in 70 patients with chronic tonsillitis, and in 144 healthy individuals (control group) and the associations between EBV infection and clinicopathological and demographic characteristics and the use of stimulants in all study groups. The objective of this study was also to analyze the prevalence of coinfection with human papillomavirus (HPV). After prior DNA isolation, EBV detection was performed using an EBV kit by real-time polymerase chain reaction. The prevalence of EBV infection in patients with HNSCC, patients with chronic tonsillitis, and the control group was 47.7%, 60%, and 24.3%, respectively. Compared to controls, a significantly higher prevalence of EBV in patients with chronic tonsillitis and HNSCC may suggest that EBV is a potential risk factor. No association was found between EBV infection and demographic or clinical data. Further studies are warranted due to inconclusive reports that were mainly related to geographic distribution, sample type, and detection technique. Considering the prevalence of the virus and the risk of serious diseases, attention should be paid to screening diagnosis and prevention of the infection.

## 1. Introduction

Epstein-Barr virus belongs to the gammaherpesviruses and is composed of an icosahedral capsid of more than 100 nm in diameter, consisting of 162 capsomeres, which envelops a double-stranded deoxyribonucleic acid (DNA) molecule [1]. Moreover, there are two EBV types, namely, EBV-1 and EBV-2 (previously known as EBV-A and EBV-B). The virus is spread through body fluids, mainly saliva, blood, and semen [2]. The symptoms of EBV infection may vary, depending on age and immunity. They are mostly mild benign in younger individuals [3]. According to various sources, 90-95% of the adult population is infected with EBV, regardless of socioeconomic status and place of residence. Recently, interest in EBV has increased significantly due to the reports on the potentially oncogenic nature of the virus [4, 5]. Studies showed that EBV could be involved in initiating and/or enhancing epithelial-mesenchymal transition (EMT), which is important in cancer progression and metastasis [6–9]. It has been reported that among oncogenic viruses, EBV may be associated with the development of tumors in head and neck squamous cell carcinoma (HNSCC) [10].

HNSCC is a disease related to important anatomical regions such as the oral cavity, the nasopharyngeal cavity, and the larynx [11]. HNSCC is the sixth most prevalent cancer worldwide (890,000 new cases and approximately 450,000 deaths annually) [12]. About 90% of all cancers of the head and neck region originate from squamous cells [13]. The occurrence of these cancers is strongly related to environmental factors and lifestyle. Studies have shown that increased risk factors for HNSCC include tobacco exposure and alcohol abuse [14–16].

The term “chronic tonsillitis” is not clearly defined, and it usually refers to a sore throat lasting more than 3 months accompanied by the inflammation of the tonsils with lymph node reaction [17]. Most chronic inflammation occurs in the palatine and/or pharyngeal tonsils [18]. Inflamed tonsils are a focus of infection that can have a significant impact on other organs. In chronic inflammation, tonsillectomy is usually performed [19].

The aim of the study was to analyze the prevalence of EBV DNA in patients with HNSCC, in patients with chronic tonsillitis, and in healthy individuals and the associations between EBV infection and clinicopathological and demographic characteristics and the use of stimulants in all study groups. The objective of this study was also to analyze the prevalence of coinfection with human papillomavirus (HPV).

## 2. Material and Methods

### 2.1. Study Population and Specimen Collection

The study was approved by the Bioethics Committee (Institutional Review Board on Medical Ethics, No. KNW/0022/KB1/49/16 and No. KNW/0022/KB1/49/II/16/17). All volunteers gave written informed consent before participating in the study.

The study was comprised of 300 patients, including 86 subjects with HNSCC and 70 patients with chronic tonsillitis. The control group consisted of 144 healthy individuals. Information on demographic variables including age, sex, and the use of stimulants such as alcohol and tobacco was collected from all patients and healthy individuals. Patients in the study groups were diagnosed in the Department of Otorhinolaryngology and Oncological Laryngology in Zabrze, Medical University of Silesia in Katowice. Cancer samples were obtained during surgical resection and tonsillar tissue samples during tonsillectomy.

The diagnosis of HNSCC in the tumor tissues was made by routine histological assessment after surgical resection. In the group with chronic tonsillitis, the samples were histologically verified by a pathologist after sampling during tonsillectomy. The samples from both groups were stored at -80°C until further analysis after being frozen in liquid nitrogen.

Buccal epithelial scrapings were collected from healthy volunteers. The oral mucosa of cheeks was scraped with a cotton swab stick followed by the DNA extraction. All molecular analyses were performed at the Department of Medical and Molecular Biology, Faculty of Medical Sciences in Zabrze, Medical University of Silesia. Moreover, the HPV infection status of all study participants was obtained from our previous study to analyze coinfections [20].

### 2.2. Study Groups

The HNSCC group consisted of 86 patients (mean age 57.9 ± 11.7 years); this group consisted of 28 (32.6%) women and 58 (67.4%) men (Table 1). In 86 patients, the anatomical sites of tumors were as follows: the tongue (*n* = 42, 48.8%), the floor of the mouth (*n* = 16, 18.6%), the mandible (*n* = 15, 17.4%), the left palatine tonsil (*n* = 7, 8.1%), the cheek and the soft palate (*n* = 6, 7.0%), and the right palatine tonsil (*n* = 5, 5.8%). One case of HNSCC was found in the retromolar trigone (1.2%). In addition, in 22 patients (25.6%), tumors were detected in more than one location. Radiation therapy or chemotherapy was not administered to any patient prior to tumor excision surgery. The clinical stage of tumors was characterized according to the TNM classification of the American Joint Committee on Cancer (AJCC) 8^th^ edition [21]. The detailed clinical data, including T-classification (T), lymph node status (N), and histological grade (G), are given in Table 2.

The chronic tonsillitis group was composed of 70 patients (mean age 33.3 ± 11.2 years); this group included 36 (51.4%) women and 34 (48.6%) men (Table 1).

The healthy control group consisted of 144 individuals (mean age 40.8 ± 16.6 years); this group was composed of 91 (63.2%) women and 53 (36.8%) men (Table 1).

### 2.3. DNA Preparation

All tumor and tonsillar tissue samples were slowly thawed and homogenized with Lysing Matrix A ceramic beads in FastPrep®-24 (MP Biomedicals, Irvin, California, USA), and then, DNA was isolated using a Gene Matrix Tissue DNA Purification Kit (EURx, Gdańsk, Poland) according to the standard procedures. In case of a control group, DNA was isolated from buccal epithelial cells using a GeneMATRIX Swab-Extract DNA Purification Kit (EURx, Gdańsk, Poland) according to the manufacturer's instructions. The quantity and quality of the extracted DNA were analyzed using a spectrophotometer (NanoPhotometer Pearl, Implen, Germany).

### 2.4. EBV Detection by Polymerase Chain Reaction

EBV detection was performed with EBV PCR Kit (EBV/ISEX/100, GeneProof, Brno, Czech Republic) using real-time PCR according to the manufacturer's protocol using QuantStudio 5 (Thermo Fisher Scientific, Waltham, Massachusetts, USA). The method consists in amplifying a specific conserved DNA sequence of a single copy gene encoding nuclear antigen 1 (EBNA1) and measuring the increase in fluorescence. The presence of EBV is indicated by the increase in fluorescence of the FAM fluorophore. An internal standard (IS) was included in the reaction mixture, which also controls the quality of the DNA extraction process. Positive IS amplification was detected in the fluorescence channel of the HEX fluorophore.

### 2.5. HPV Detection and HPV Type Determination

HPV infection status of all study participants was obtained from our previous study [20]. HPV detection and identification of subtypes were performed by GenoFlow HPV (Human Papillomavirus) Array Test Kits (DiagCor Bioscience Inc., Hong Kong). Colored dots on the membrane indicated positivity and were recorded by scanning (CapturePro Image, CaptureREAD 3.1.; DiagCor Bioscience Inc., Hong Kong). All runs included positive and negative controls and amplification and hybridization controls.

### 2.6. Statistical Analysis

The Kruskal-Wallis test and the Fisher exact test were used to compare the groups based on the EBV status and age, sex, smoking, occasional/regular alcohol consumption, and both alcohol and tobacco use. The EBV status and EBV and HPV coinfection impact on clinical T-classification (T), lymph node status (N), histological grade (G), and localization of the primary tumor were assessed using the Kruskal-Wallis and Fisher exact tests. All statistical analyses were performed using the STATISTICA 13.3 software (StatSoft. Inc., Tulsa, Oklahoma, USA) with the significance level of *α* < 0.05 for complete sets of clinical data. Therefore, some patients or controls were excluded from the study.

## 3. Results

### 3.1. The Prevalence of EBV in All Study Groups

**Figure 1:**
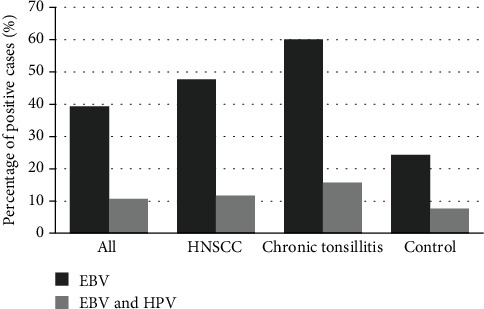
Percentage of positive EBV cases and coinfection of EBV and HPV in all study groups.

EBV was present in 60% (42/70) of patients with chronic tonsillitis, which was significantly higher (*p* < 0.05) than in the control group (24.3%, 35/144). However, it was not significantly (*p* = 0.13) more prevalent than in the HNSCC group (47.7%, 41/86). EBV-positive status was significantly more prevalent in the HNSCC group and chronic tonsillitis group than in the healthy control group (*p* < 0.05, Figure 1).

Coinfection of HPV and EBV was the most prevalent in the group with chronic tonsillitis (15.7%). In the HNSCC group, coinfection occurred in 11.6% of patients while in the control group only in 7.6% (Figure 1). Coinfection of EBV with HPV was significantly higher in HNSCC patients with lymph node status N2 (Table S1). This effect could only be observed in patients with coinfection. No other significant differences were observed in terms of the prevalence of coinfection.

### 3.2. Clinical and Demographic Data, the Use of Stimulants, and the EBV Status in the Groups with HNSCC and Chronic Tonsillitis and in the Control Group

No significant association was found between the EBV status and age, sex, smoking, occasional/regular alcohol consumption, or both alcohol consumption and smoking in groups with HNSCC and chronical tonsillitis or in the control group. Additionally, no significant association was found between the EBV status and clinical T-classification (T), lymph node status (N), histological grade (G), or the location of the primary tumor in HNSCC patients. When analyzing all samples (*n* = 300), we found only one significant association, which was the lower prevalence of EBV infections in the group of occasional and regular alcohol users (Table S2).

## 4. Discussion

Epstein-Barr virus (EBV) is the first herpesvirus to be linked to cancer and is known to infect most of the adult human population. The EBV life cycle is divided into a lytic phase and a latent phase. Expression of latent EBV genes in infected epithelial cells is probably associated with their transformation into cancer cells [22].

The aim of the study is to clarify whether EBV has any impact on the biological behaviour of HNSCC carcinogenesis and chronic inflammation process by analyzing EBV prevalence and clinicopathological and demographic characteristics in HNSCC patients, in patients with chronic tonsillitis, and in healthy individuals. Our analysis also focuses on external environmental factors, such as alcohol use and cigarette smoking, and coinfection with human papillomavirus (HPV).

Polz-Gruszka et al. [23] conducted a study on a Polish population and reported that EBV was detected in 57.5% (46/80) of samples, including 60% (30/50) of laryngeal carcinomas and 53.3% (16/30) of oropharyngeal carcinomas. In addition, the virus has been reported more frequently in patients over 50 years of age (66.7%) [24]. In another study by Polz-Gruszka et al. [24], which included 154 patients with primary oral squamous cell carcinoma (OSCC) and oropharyngeal squamous cell carcinoma (OPSCC) in the Polish population, it was shown that 27.3% of all tested specimens were positive for EBV, in oropharyngeal 29.1%, and in oral cavity 26.1% [24]. In a study involving 78 patients with histologically confirmed OPSCC and 40 healthy controls from Poland, EBV DNA was detected in 51.3% of tumor samples and 20% of controls [25]. In a subsequent study based on 146 Polish patients, the prevalence of EBV infection was 34.3% in oropharyngeal cancer and 8.6% in cancer of the oral cavity [26]. In our study, EBV was present in 47.7% of cases in HNSCC. Such different results of EBV detection may be related to the differences in the HNSCC groups, particularly connected with various locations of primary tumors. She et al. conducted an important meta-analysis to examine the association of EBV with OSCC [27]. The results of 13 studies [28–40] suggested a positive association between EBV infection and the risk of OSCC. The total number of participants was 686 patients and 433 controls. Nine studies used paraffin-embedded tissues and four studies used fresh frozen tissues. Different detection techniques such as PCR, nested PCR, RT-qPCR, IHC (immunohistochemistry), and ISH (*in situ* hybridization) were used to detect EBV [27]. These tests showed differential detection of EBV. Therefore, adopting adequate methodology and techniques is crucial in the detection of EBV. There is still no gold standard in this type of research. Moreover, data suggests that the role of EBV in OSCC might depend on the geographical location [41]. Jaloluli et al. [42] detected EBV in the samples of OSCC from 8 different countries (Norway, UK, Sweden, USA, Sri Lanka, India, Sudan, and Yemen). Of the 155 oral carcinomas, 85 (55%) were positive for EBV. EBV-positive samples (80%) were the most prevalent among the UK patients.

In our study in HNSCC cases, coinfection of EBV and HPV occurred in 11.6% of patients. Moreover, coinfection was associated with pathoclinical features. In the group of patients with the lymph node status of N2, coinfection of EBV and HPV was observed more frequently. In a study by Polz-Gruszka et al. [24] conducted on the Polish population, coinfection with EBV and HPV was detected in 15% of samples [24]. In another study by Polz-Gruszka et al. [24], which was conducted on the Polish population of 154 patients with primary OSCC and OPSCC, the coinfection of HPV and EBV was detected in 7.8% (12/154) of the patients, 9.7% (6/62) among oropharyngeal patients, and 6.5% (6/92) among oral cavity patients. No correlation was found between HPV or EBV infection and age or tobacco smoking [24]. In another study, which was aimed at analyzing the prevalence of coinfection with HPV, EBV, and polyoma BK virus (BKPyV) in oral, oropharyngeal, and laryngeal squamous cell carcinomas in 146 Polish patients, a single infection was detected in 43.8% of the samples and mixed infections in 56.2%. Coinfections for HPV/EBV, HPV/BKV, and EBV/BKV were detected in 34.1%, 22%, and 23.2% of samples, respectively. All three tested viruses were present in 17 specimens. HPV was more often detected in oral cavity cancer patients (44.5%) while EBV in oropharyngeal cancer (57.1%). EBV was also detected in laryngeal cancer (34.3%) and in oral cavity cancer (8.6%) patients. Grade G3 and stages N1 and N2 were the most common in patients with HPV/EBV coinfection. Stages N1 and N2 were characteristic of infection with all three viruses. Grade G3 was present four times higher in HPV/EBV coinfection, five times higher in EBV/BKV coinfection, and ten times higher in samples with all three viruses in contrast to only EBV detection [26]. Jiang et al. suggested that the presence of EBV infection may promote the invasiveness of HPV-positive OPSCC tumors [43]. On the other hand, the presence of HPV may increase the pathogenic effects of EBV in the oral cavity by affecting epithelial cells and prolonging EBV cell cycle [44]. Contrary to that study, Guidry et al. [45] showed a change in the life cycle of EBV after infection with HPV of the immortalized epithelial tissue. HPV-related cell changes contributed to the formation of a latent EBV infection [45].

Some authors showed that EBV was also detected in tonsillar diseases, including chronic tonsillitis [46–50]. In our study population, we observed statistically significant differences in the incidence of EBV-positive cases between the group with chronic tonsillitis and the control group (*p* < 0.05; 60% vs. 24.3%). Some authors indicated a differential frequency of the virus in recurrent tonsillitis and hypertrophic tonsillitis ranging from 26 to 43% [48, 51–54]. It is reported that the prevalence of EBV associated with tonsillitis varied depending on the method [48]. Higher detection of EBV in tonsillar tissues was demonstrated in studies using PCR, which is considered a rapid, specific, and sensitive method to detect minimal amount of viral DNA as reported by Peiper et al. [55]. Using this method, our study showed the presence of EBV in the tonsillar tissues of patients with chronic tonsillitis in 42/70 (60%) cases. Similarly, other studies related to chronic tonsillitis indicated the presence of EBV in 54.1% of cases (13/24) [47] and in 46% of cases (23/50) [49]. Other reports of noncancerous tonsillitis detected the virus in most cases, i.e., in 53.6% [56] and 58% in chronic hyperplasic tonsillitis [57] and in 64% in tonsils of acute infectious mononucleosis [58]. It is suggested that oral and pharyngeal epithelial cells, particularly the tonsillar tissue, are considered the main reservoir of EBV [48, 57]. Furthermore, other studies suggested tonsil lymphocytes as the site of virus replication [49, 50, 53].

Gonzalez-Lucano [49] reported that younger age of patients with chronic tonsillitis was associated with higher presence of EBV [49]. Such reports were also confirmed by other studies on noncancerous tonsillar tissue, such as recurrent and hypertrophic tonsillitis [46, 48, 51]. It is suspected that EBV may infect the tonsils of children and thus contribute to the development of recurrent and hypertrophic tonsillitis [48]. Another hypothesis is that overuse of antibiotics leads to changes in the tonsillar microbiome and increases the risk of viral infections of tonsils [47]. In our study, we did not observe a correlation between the age of patients with chronic tonsillitis and an increased prevalence of EBV. In our study group, it can be explained by the absence of children, who usually have high EBV copy number. However, time and maturation of virus-specific immunity are essential factors in suppression [56].

In addition, our study showed 15.7% EBV/HPV coinfection in the chronic tonsillitis group. A search of databases (PubMed and Medline) revealed no other reports suggesting coinfection in chronic tonsillitis. Xue et al. who examined samples of children with tonsillar or adenoid hypertrophy demonstrated EBV infection in 45% of tonsillar tissues but found no EBV/HPV coinfection in any patient [59]. Similar results were obtained by Jiang [43], who observed EBV infection in 20% of noncancer base of tongue samples. However, HPV was not confirmed [43]. In a study of patients with nonmalignant tonsils, of the above viruses, only DNA-EBV was detected in more than half of the samples. The authors concluded that HPV and EBV infected independently of each other [60]. Interestingly, *in vitro* studies suggest that in the case of coinfection in oral squamous epithelial cells, the presence of HPV may increase the pathogenesis of EBV [44].

To conclude, it seems that the etiology of chronic tonsillitis can be related to EBV. Further research should focus on explaining the role of EBV infection in chronic tonsillitis in a larger population. The data could be useful in designing public health strategies, including clinical examination and EBV testing.

The prevalence of EBV in healthy individuals ranges from 0 to 90%. In our case-control study, we detected 24.3% (35/109) cases of EBV in oral swab specimens, which is in line with other reports in which EBV was detected in 18.1% (17/94) of samples of exfoliated oral cells [61], in 22% (34/157) of throat washings [62], and in 20% (4/20) of samples of squamous epithelial cells collected by scraping [63]. EBV DNA was found in saliva in more than 30% of cases [64, 65], which is in line with Mao and Smith [66] who reported that 25% (15/60) of nonsmokers and occasional alcohol users showed EBV positivity [66]. No association was found between the EBV status and demographic factors or the use of stimulants, including smoking and drinking alcohol, which is in line with other studies [66, 67]. However, Kuri et al. reported more frequent EBV seropositivity in women [68]. Mao and Smith [66] showed a statistically insignificant relationship between older age (>60 years) and the prevalence of EBV [66]. Several studies found that the prevalence of EBV increased with age [64, 68]. Contrary to these reports, Gupta et al. observed EBV most frequently in the age group of 30-39 years [69]. Another study reported no effect of older age on the prevalence of EBV [38]. The virus was also detected in serum samples in 61% [69] and 85.3% of cases [68]. Significantly higher results were shown in a study in which the virus was detected in 90% (43/48) of throat washings of adult patients, which coincides with the overall data on EBV seropositivity in the Japanese population [64].

In our study, we detected viral coinfection in 7.6% of healthy volunteers. A similarly low result of several percent was obtained by McCormick et al. (13.3%) [70] and Lattario et al. (15%) [71] for cervical samples from healthy individuals. In a Polish study, the authors reported HPV (2.5%) and EBV (20%) infection in serum and saliva samples [25]. In the Tunisian study by Kahla et al., no HPV or EBV infection was detected in cervical biopsies of the control group [72]. Further research into the mechanisms related to HPV and EBV cooccurrence, as well as screening of healthy individuals for early detection of oncogenic viruses that affect the risk of cancer progression, is important [72].

Rapid advances in scientific fields such as molecular biology, genetic engineering, and virology have contributed to the development of new vaccine options, both for the prevention of primary infections and subsequent chronic diseases. Because EBV is related with many malignancies including HNSCC, development of EBV vaccine is also required.

## 5. Conclusion

In conclusion, the prevalence of EBV in HNSCC and chronic tonsillitis was significantly higher than in the control group, which suggests that EBV may be defined as a potential risk factor for these diseases. The findings showed that oncogenic viruses, including EBV, are not the only risk factors in the development of HNSCC but may promote carcinogenesis with other etiological factors. Considering the prevalence of the virus and the risk of a serious disease, attention should be paid to screening diagnosis and prevention of the infection. In addition to the need for widely available rapid methods to effectively detect EBV, a prophylactic EBV vaccine would be promising in the prevention of EBV-associated cancers.

## Figures and Tables

**Table 1:** Summary of the main characteristics of the study participants.

		HNSCC	Chronic tonsillitis	Control	All
		*n* (%)	*n* (%)	*n* (%)	*n* (%)
Sex	Female	28 (32.56%)	36 (51.43%)	91 (63.19%)	155 (51.67%)
	Male	58 (67.44%)	34 (48.57%)	53 (36.81%)	145 (48.33%)
Smoking	Yes	59 (69.41%)	10 (14.29%)	25 (17.36%)	94 (31.44%)
	No	26 (30.59%)	60 (85.71%)	119 (82.64%)	205 (68.56%)
Occasional drinking	Yes	56 (65.12%)	21 (30%)	107 (74.31%)	184 (61.33%)
	No	30 (34.88%)	49 (70%)	37 (25.69%)	116 (38.67%)
Regular drinking	Yes	42 (48.84%)	2 (2.86%)	10 (6.94%)	54 (18%)
	No	44 (51.16%)	68 (97.14%)	134 (93.06%)	246 (82%)
Both smoking and drinking	Yes	43 (50%)	6 (8.57%)	23 (15.97%)	72 (24%)
	No	43 (50%)	64 (91.43%)	121 (84.03%)	228 (76%)

**Table 2:** Clinical data of the HNSCC group.

	*n*	(%)
Clinical T-classification	86	(100.00)
T1	10	(11.6)
T2	22	(25.6)
T3	26	(30.2)
T4	28	(32.6)
T1+T2	32	(37.2)
T3+T4	54	(62.8)
Lymph node status	86	(100.00)
N0	39	(43.3)
N1	21	(24.4)
N2	23	(26.7)
N3	3	(3.5)
N1+N2	44	(51.2)
Histological grade	86	(100.00)
G1	13	(15.1)
G2	54	(62.8)
G3	19	(22.1)
G1+G2	66	(76.7)

## Data Availability

The data used to support the findings of this study are available from the corresponding author upon request.

## Abbreviations

AJCC: American Joint Committee on Cancer
BNLF: Brain-derived neurotrophic factor
EBERs: Epstein-Barr virus-encoded small RNAs
EBNA: Epstein-Barr nuclear antigen
EBV: Epstein-Barr virus
EMT: Epithelial-mesenchymal transition
HHV-4: Human gammaherpesvirus 4
HIV: Human immunodeficiency viruses
HNSCC: Head and neck squamous cell carcinoma
HPV: Human papillomavirus
IHC: Immunohistochemistry
ISH: *In situ* hybridization
LMP: Epstein-Barr virus latent membrane protein
miRNA: MicroRNA
NPC: Nasopharyngeal carcinoma
OPSCC: Oropharyngeal squamous cell carcinoma
OSCC: Oral squamous cell carcinoma
PCR: Polymerase chain reaction
PTEN: Phosphatase and tensin homolog
STAT3: Signal transducer and activator of transcription 3
TNBC: Triple-negative breast cancer.

## Supplementary Materials

Supplementary 1 Table S1: association of EBV infection and coinfection of EBV and HPV and clinical parameters.

Supplementary 2 Table S2: association between EBV infection and coinfection of EBV and HPV and demographic parameters and smoking and alcohol consumption.
