# Supplementary material for: PCR Detection of Epstein-Barr Virus (EBV) DNA in Patients with Head and Neck Squamous Cell Carcinoma, in Patients with Chronic Tonsillitis, and in Healthy Individuals
Source: Biomed Res Int. 2022 Aug 8;2022:8506242. doi: 10.1155/2022/8506242 (PMC9381848; doi:10.1155/2022/8506242)
Supplement: Supplementary 1 — Table S1: association of EBV infection and coinfection of EBV and HPV and clinical parameters. [file 8506242.f1.docx]

**Table S1.** Association of EBV infection and co-infection of EBV and HPV and clinical parameters. Fisher exact test p value (p), odds ratio (OR) and odds ratio 95% confidence intervals (ORCI) for clinical T-classification (T), lymph node status (N), histological grade (G) in HNSCC group.

| HNSCC group |  | EBV |  |  | Co-infection of EBV and HPV | | |
| --- | --- | --- | --- | --- | --- | --- | --- |
|  |  | p | OR | ORCI | p | OR | ORCI |
| T-classification (T) | T1 | 0.741 | 1.42 | 0.37-5.45 | 1.000 | 1.81 | 0.21-15.41 |
|  | T2 | 1.000 | 1.13 | 0.43-2.98 | 0.270 | 0.47 | 0.12-1.83 |
|  | T3 | 0.639 | 1.36 | 0.54-3.45 | 0.482 | 0.61 | 0.16-2.38 |
|  | T4 | 0.255 | 0.57 | 0.23-1.41 | 0.488 | 2.08 | 0.41-10.51 |
|  | T1+T2 | 0.657 | 1.29 | 0.53-3.1 | 1.000 | 0.88 | 0.23-3.37 |
|  | T3+T4 | 0.657 | 0.78 | 0.32-1.87 | 1.000 | 1.14 | 0.3-4.4 |
| lymph node status (N) | N0 | 0.135 | 1.98 | 0.83-4.7 | 1.000 | 1.28 | 0.33-4.91 |
|  | N1 | 1.000 | 1.00 | 0.37-2.69 | 0.284 | 4.32 | 0.53-35.49 |
|  | N2 | 0.341 | 0.62 | 0.24-1.61 | **0.020** | **0.19** | **0.05-0.76** |
|  | N3 | 0.189 | 0.21 | 0.02-1.98 | 0.520 | 0.59 | 0.06-5.82 |
|  | N1+N2 | 0.398 | 0.68 | 0.29-1.6 | 0.739 | 0.67 | 0.17-2.55 |
| histological grade (G) | G1 | 0.766 | 0.75 | 0.23-2.44 | 0.172 | 0.35 | 0.08-1.6 |
|  | G2 | 1.000 | 0.95 | 0.4-2.28 | 0.490 | 1.81 | 0.48-6.83 |
|  | G3 | 0.613 | 1.33 | 0.48-3.73 | 1.000 | 1.15 | 0.22-5.95 |
|  | G1+G2 | 0.804 | 0.87 | 0.32-2.37 | 0.692 | 1.49 | 0.35-6.38 |
