# Supplementary material for: PCR Detection of Epstein-Barr Virus (EBV) DNA in Patients with Head and Neck Squamous Cell Carcinoma, in Patients with Chronic Tonsillitis, and in Healthy Individuals
Source: Biomed Res Int. 2022 Aug 8;2022:8506242. doi: 10.1155/2022/8506242 (PMC9381848; doi:10.1155/2022/8506242)
Supplement: Supplementary 2 — Table S2: association between EBV infection and coinfection of EBV and HPV and demographic parameters and smoking and alcohol consumption. [file 8506242.f2.docx]

**Table S2.** Association between EBV infection and co-infection of EBV and HPV and demographic parameters and smoking and alcohol consumption. Fisher exact test p value (p), odds ratio (OR) and odds ratio 95% confidence intervals (ORCI) within study groups.

|  |  | EBV | | | Co-infection of HPV and EBV | | |
| --- | --- | --- | --- | --- | --- | --- | --- |
|  |  | p | OR | ORCI | p | OR | ORCI |
| HNSCC | Sex | 1.000 | 1.08 | 0.44-2.66 | 1.000 | 1.14 | 0.27-4.8 |
|  | Smoking | 1.000 | 0.95 | 0.38-2.39 | 0.271 | 2.57 | 0.67-9.8 |
|  | Occasional alcohol consumption | 0.501 | 1.42 | 0.58-3.45 | 0.090 | 3.25 | 0.84-12.59 |
|  | Regular alcohol consumption | 0.398 | 1.46 | 0.62-3.42 | 0.739 | 1.50 | 0.39-5.74 |
|  | Smoking and drinking | 0.666 | 1.32 | 0.57-3.09 | 0.313 | 2.59 | 0.62-10.78 |
|  | No stimulant use | 1.000 | 1.07 | 0.33-3.51 | **0.040** | **0.20** | **0.05-0.85** |
| Chronic tonsillitis | Sex | 0.626 | 0.72 | 0.27-1.87 | 0.515 | 0.55 | 0.15-2.09 |
|  | Smoking | 0.296 | 0.33 | 0.06-1.67 | 0.186 | 0.36 | 0.08-1.68 |
|  | Occasional alcohol consumption | 1.000 | 0.89 | 0.31-2.55 | 0.723 | 0.71 | 0.18-2.74 |
|  | Regular alcohol consumption | 0.642 | 0.47 | 0.05-4.76 | 0.292 | 0.17 | 0.01-2.99 |
|  | Smoking and drinking | 1.000 | 0.73 | 0.12-4.29 | 1.000 | 0.93 | 0.1-8.79 |
|  | No stimulant use | 0.445 | 1.70 | 0.61-4.74 | 0.183 | 2.53 | 0.68-9.33 |
| Control | Sex | 0.110 | 1.92 | 0.88-4.15 | 1.000 | 0.98 | 0.27-3.52 |
|  | Smoking | 1.000 | 1.02 | 0.37-2.8 | 1.000 | 0.94 | 0.19-4.65 |
|  | Occasional alcohol consumption | 0.661 | 1.21 | 0.52-2.85 | 1.000 | 1.09 | 0.27-4.35 |
|  | Regular alcohol consumption | 0.705 | 0.73 | 0.18-3 | 0.560 | 0.73 | 0.08-6.32 |
|  | Smoking and drinking | 0.796 | 0.89 | 0.32-2.48 | 0.689 | 0.84 | 0.17-4.19 |
|  | No stimulant use | 0.504 | 0.74 | 0.32-1.76 | 0.729 | 0.84 | 0.21-3.38 |
| All | Sex | 0.124 | 1.48 | 0.93-2.35 | 1.000 | 0.94 | 0.45-1.95 |
|  | Smoking | 0.203 | 0.71 | 0.43-1.17 | 1.000 | 1.01 | 0.46-2.23 |
|  | Occasional alcohol consumption | **0.029** | **1.73** | **1.08-2.78** | 0.182 | 1.68 | 0.8-3.51 |
|  | Regular alcohol consumption | 0.645 | 0.85 | 0.47-1.54 | 1.000 | 0.95 | 0.37-2.42 |
|  | Smoking and drinking | 0.890 | 0.95 | 0.55-1.63 | 0.521 | 1.42 | 0.56-3.59 |
|  | No stimulant use | 0.307 | 0.75 | 0.46-1.23 | 0.422 | 0.72 | 0.34-1.55 |
